# Supplementary material for: Reliability of center of pressure excursion as a measure of postural control in bipedal stance of individuals with intellectual disability: A pilot study
Source: PLoS One. 2020 Oct 21;15(10):e0240702. doi: 10.1371/journal.pone.0240702 (PMC7577434; doi:10.1371/journal.pone.0240702)
Supplement: S1 Table — (DOCX) [file pone.0240702.s001.docx]

**S1 Table. Summary of stabilometric studies in static bipedal stance on a sample that includes adolescents and adults with intellectual disability (ID).**

| **Study** | **ID sample** | **IQ** | **Age (years)** | **Sex (M / F)** | **Sampling frequency (Hz)** | **Trial length (s)** | **Rep** | **Experimental conditions** | **COP parameters reported** |
| --- | --- | --- | --- | --- | --- | --- | --- | --- | --- |
| Ko et al, 1992 [1] | 32 ID | mild to profound | *M* = 36  range = 21–62 | NR | NR | 10 | 2 | 1. EO, arms on side 2. EO, arms raised forward 3. EO, arms swaying | Sway area  Path length  Sway amplitude |
| Van Emmerik et al., 1993 [2] | 40 ID | severe to profound | *M* = 35  range = 21–60 | NR | 200 | 10 | 2 | 1. EO, arms on side 2. EO, right arm raised 3. EO, left arm raised 4. EO, both arms raised | Sway pattern |
| Suomi & Koceja, 1994 [3] | 22 ID | *M* = 57.8  *SD* = 9.7 | *M* = 30.3  *SD* = 5.5 | 22 / 0 | 50 | 15 | 3 | 1. EO 2. EC | Sway amplitude |
| Carmeli et al., 2008 [4] | 15 ID | mild | *M* = 44.4  *SD* = 5.39 | 5 / 10 | 200 | 15 | NR | 1. EO, pendular arm movement 2. EC, pendular arm movement 3. EO, no pendular arm movement 4. EC, no pendular arm movement | Sway velocity  Sway amplitude  Weight distribution |
| Dellavia et al., 2009 [5] | 30 DS with ID  30 ID (non-specific) | NR | *M* = 26.9  *SD* = 6.2  *M* = 29.9  *SD* = 6.3 | 15 / 15  15 / 15 | NR | 40 | 1 | 1. EO, no added oral proprioceptive input 2. EC, no added oral proprioceptive input 3. EO, added oral proprioceptive input 4. EC, added oral proprioceptive input | Sway area |
| Blomqvist et al., 2013 [6] | 100 ID | mild to moderate | *M* = 17.9  *SD* = 1.2 | 60 / 40 | 200 | 30 | 3 | 1. EO, feet together 2. EC, feet together 3. EO, tandem stance 4. EC, tandem stance 5. EO, left single leg stance 6. EC, left single leg stance 7. EO, right single leg stance 8. EC, right single leg stance | Sway velocity |
| Zur et al., 2013 [7] | 21 ID | *M* = 57.9  *SD* = 6.7 | *M* = 17.5  *SD* = 3.9 | 18 / 3 | 100 | 30 | 5 | 1. EO, tandem stance 2. EC, tandem stance | Sway area  Sway velocity  Sway range |

IQ, intelligence quotient; Rep, repetition; COP, center of pressure; EO, eyes open; EC, eyes closed; NR, not reported

***References***

1. Ko YG, Van Emmerik RE, Sprague RL, Newell KM. Postural stability, tardive dyskinesia, and developmental disability. J Intellect Disabil Res. 1992;36:309–23. doi:10.1111/j.1365-2788.1992.tb00530.x
2. Van Emmerik RE, Sprague RL, Newell KM. (1993). Quantification of postural sway patterns in tardive dyskinesia. Mov Disord. 1993;8:305–14. doi:10.1002/mds.870080309
3. Suomi R, Koceja DM. Postural sway patterns of normal men and women and men with mental retardation during a two-legged stance test. Arch Phys Med Rehabil. 1994;75:205–9. doi:10.1016/0003-9993(94)90397-2
4. Carmeli E, Bar-Yosef T, Ariav C, Paz R, Sabbag H, Levy R. Sensorimotor impairments and strategies in adults with intellectual disabilities. Motor Control. 2008;12:348–61. doi:10.1123/mcj.12.4.348
5. Dellavia C, Pallavera A, Orlando F, Sforza C. Postural stability of athletes in Special Olympics. Percept Mot Skills, 2009;108:608–22. doi:10.2466/PMS.108.2.608-622
6. Blomqvist S, Olsson J, Wallin L, Wester A, Rehn B. Adolescents with intellectual disability have reduced postural balance and muscle performance in trunk and lower limbs compared to peers with intellectual disability. Res Dev Disabil. 2013;34:198–206. doi:10.1016/j.ridd.2012.07.008
7. Zur O, Ronen A, Melzer I, Carmeli E. Vestibulo-ocular response and balance control in children and young adults with mild-to-moderate intellectual and developmental disability: A pilot study. Res Dev Disabil. 2013;34:1951–7. doi:10.1016/j.ridd.2013.03.007
