# Supplementary material for: Reliability of center of pressure excursion as a measure of postural control in bipedal stance of individuals with intellectual disability: A pilot study
Source: PLoS One. 2020 Oct 21;15(10):e0240702. doi: 10.1371/journal.pone.0240702 (PMC7577434; doi:10.1371/journal.pone.0240702)
Supplement: Attachment — Submitted filename: Response_to_Reviewers.docx [file pone.0240702.s003.docx]

PLOS ONE

**REBUTTAL LETTER**

**[PONE-D-20-19187]** Reliability of center of pressure excursion as a measure of postural control

in bipedal stance of individuals with intellectual disability: A pilot study

First of all, we would like to express our gratitude to the reviewers who have taken the time to review our manuscript for possible publication in PLOS ONE. We have carefully reviewed your comments and responded to them with the aim of improving our manuscript. To aid the reviewers, we prepared a matrix to organize our responses to the comments given by the reviewers. We have numbered all the comments of both reviewers for ease of reference, as can be seen in first column of the matrix. Columns 2 and 3 of the matrix indicate the verbatim comments of the reviewers and the corresponding authors’ response, respectively. The last column provides the location of the revision (in the file “Revised Manuscript with Track Changes”), if it is applicable.

**Review Comments from Reviewer # 1**

The authors of the article entitled “Reliability of center of pressure excursion as a measure of postural control in bipedal stance of individuals with intellectual disability: A pilot study” aimed to test reliability of different stabilometric protocols for individuals with intellectual disability (ID) based on optimal combinations of shortest necessary trial durations and the least number of trial repetitions that guarantee sufficient reliability. The authors found that to achieve acceptable reliability, four 30-s trials of each experimental condition appeared to be optimal for testing participants both individuals with and without ID.

The study topic is interesting and may contribute to improve postural control investigation accuracy in individuals with ID. In general, the study is well designed. However, the authors have to respond to some comments regarding the manuscript. Also, there are some sentences that require rewriting and some statements that need to be addressed and clarified.

| Comment # | Reviewer’s Comment | Authors’ Action | Location in Revision |
| --- | --- | --- | --- |
|  | Abstract: |  |  |
| 1 | Authors are encouraged to more describe the method in the abstract. If there was a word limit condition you can shorten the Background of the Abstract. | Added as advised but, indeed, some background details of the abstract had to be rephrased or deleted to keep within the 300 word count limit | p. 2, lines 23–36. |
|  | Introduction |  |  |
| 2 | I think that the use of tables to summarize literature is not appropriate in original article. | We gladly follow the two reviewers in their advice to remove the said table. Although our initial response was to argue for keeping the table in its current location, we understood the reasoning behind both reviewers’ comment to omit it. Keeping in mind that it could still benefit readers who may appreciate a more detailed comparison of methodological variation between stabilometric studies in ID-participants, we suggest, if it is possible, to move the table as a supplemental material. The use of such a table in the introduction is not necessarily unique to our paper. Articles by Meshkati et al.^[1]^ and Smolders et al.^[2]^ provided tables that strongly supported the rationale for their respective studies. | p. 4, lines 107–110  *S1 table added to the submission* |
| 3 | Line 64: Because this also has consequences on the reliability of results gained from stabilometry, our current study aimed to develop a reliable stabilometric protocol for evaluating postural control of adults with ID. This sentence needs to be reworded. | Rephrased as advised | p. 3, lines 76–78 |
| 4 | Line 73: This applies to individuals with ID who may fatigue more easily [15] and have limited attention and motivation to complete tasks [16]. This sentence needs to be reworded. | Rephrased as advised | p. 4, lines 104–105 |
| 5 | Line 92: please replace with by including | The line where this phrase was situated has been deleted in this revision |  |
|  | Method |  |  |
| 6 | Postural control may be influenced by sex. So, including both male and female could be the origin of high standard deviation. | This aspect has been considered during the recruitment. However, the screening for exclusion criteria precluded participation of several ID-males. We decided to keep the only female ID-participant owing to the already low sample size of this pilot study. We have added sex heterogeneity of the group under study limitations. | p. 18, lines 527–528 |
| 7 | Postural control may be influenced also by the foot size. Please provide information. | Added mean and standard deviation of participants’ foot length in the table of participant description, as advised. | p. 10, Table 1, row 4 |
| 8 | Furthermore, the groups were not BMI matched that could influence results. | Indeed BMI has been shown to influence postural control. While it is ideal to include BMI in participant matching, we also considered the high prevalence of overweight and obesity in the ID-population^[3]^ and the number of postural control studies^[4]^ with ID-participants that have similarly reported group difference in BMI between ID- and non-ID sample. |  |
| 9 | For individuals with ID, authors must obtain written informed consent from parents or legal garden. | We understand the concern you have over the capacity for consent among individuals with ID. Our institutional ethics committee (IEC) has raised this same point. The nature of their impairment raises the issue of their ability to make informed decision. However, there is reason to doubt that everyone diagnosed with ID automatically have no capacity to provide informed consent. One study from Journal of Policy and Practice in Intellectual Disability^[5]^ demonstrated that persons with ID are able to make informed decisions to participate in low risk studies. Furthermore, all of our participants, based on the information obtained from the residential home manager have legal capacity to decide on their finances and health. Thus, we believe it is an infringement of their ability to exercise personal autonomy in deciding whether to join or not if we were to require consent from parents or legal representative.  To ensure understanding of the informed consent process, we always read it out loud for them and ask at specific sections if they understand it, if they have questions about it, and if they agree to it. We also asked them to convey their understanding by having them express in their own words what the study is about, what voluntary means, and what kind of risks they will be exposed to, and what is expected of them. These measures were approved by our IEC and were enforced during data collection. | p. 6, lines 213–222 |
| 10 | Having history of injury in the past 12 month requiring medical attention must be one of exclusion criteria in postural control exploration. | This detail was under the second exclusion criteria, “neurologic, orthopedic, muscular, or cardiovascular symptoms or diagnosed medical condition.” We added another item in the exclusion list to explicitly mention this, as advised. | p. 5, lines 196–197 |
| 11 | Line 144: Please replace was by were | Replaced as advised | p. 6, line 226 |
| 12 | Line 147: We had four experimental conditions, with two vision by two surface conditions. Please rephrase this sentence; use the passive form | Rephrased as advised | p. 6, lines 229–230 |
| 13 | Line 151-153: This detail must be placed in the “Participant” section. Furthermore, if I understood correctly, you realized the IQ assessment using the Wechsler Abbreviated Scale of Intelligence. So, you must present this in the experimental design as a part of your study. | We could not place this information under the participant subsection as it is not truly an information about the participant but rather the rationale why we decided to administer an IQ test. Furthermore, we have already mentioned the IQ test administration under experimental protocol (see Line 61 of original submission) but we have added details to make this more explicit in the revision. | p. 7, lines 269–272 |
| 14 | Please specify what participants do during the one minute of rest (standing; seated). | Added information as advised | p. 7, lines 268–269 |
| 15 | Please specify if all participants have the same foot positioning on the platform and the foam surface. | Added information as advised | p. 7, line 276 |
| 16 | Please specify the characteristics of foam surface | Added information as advised | p. 6, line 232 |
|  | Results |  |  |
| 17 | Line 236: Please consider “Table 4 compares the ID- and the non-ID-group on COP parameters having acceptable reliability. | Changed as advised | p. 13, line 380 |
|  | Tables |  |  |
| 18 | All tables must be at the same form with the table 3 | Edited the tables as advised | p. 10, Table 1 (*former table 2*)  p. 13–14, Table 3 (*former table 4*) |

**Review Comments from Reviewer # 2**

Compliments to the authors for paying attention to this important topic. Balance issues are a large problem in people with ID, as well as suitable measuring instruments. This study into measures of postural control is of relevance for future studies into this topic.

Points below have to be addressed to allow the reader to better understand the choices made in the study, the procedures and some details.

| Comment # | Reviewer’s Comment | Authors’ Action | Location in Revision |
| --- | --- | --- | --- |
|  | Introduction |  |  |
| 19 | Overall, the introduction is quite long, and I think it is possible to reduce the length a bit and make it more to the point. For example, the parts about the parameters that influence reliability are informative, but because of the length deviate the attention from the relevance of this study for people with ID. I think making this part a bit more to the point will help in strengthening the introduction. | As advised, we consolidated several paragraphs about the variables affecting reliability into a single paragraph while still keeping enough details for readers unfamiliar with the topic. | p. 3–4, lines 79–85, 90–98 |
| 20 | Page 3, line 62-64: It is not clear from the text that the lack of standardization in measurement procedures is an issue in the general population or specifically in people with ID. By reading the rest of the introduction I assume both, but it is important to specify this, throughout the text. | We have clarified this part and have added a phrase to highlight this point explicitly, as advised. | p. 3, line 75–76  p. 17, line 492 |
| 21 | Page 3, line 73: based on what findings is a trial length of 30s specified for clinical populations? | Information added as advised | p. 4, lines 102–103 |
| 22 | Table 1 can be omitted from the introduction. If this was a review providing a complete overview of the studies this would be relevant, however this overview is not complete as stated by the authors. It is sufficient to describe the differences seen in test protocols in people with ID (Sampling frequency, trial length, experimental conditions, outcome parameters) in the text. | We gladly follow the two reviewers in their advice to remove the said table. Although our initial response was to argue for keeping the table in its current location, we understood the reasoning behind both reviewers’ comment to omit it. Keeping in mind that it could still benefit readers who may appreciate a more detailed comparison of methodological variation between stabilometric studies in ID-participants, we suggest, if it is possible, to move the table as a supplemental material. The lack of completeness in the overview table does not detract from our purpose, which is to demonstrate the differences in protocol that strongly supports the rationale for identifying a reliable protocol for stabilometry. Even in some types of review papers (e.g., narrative review), completeness is not necessarily a criteria to determine how useful it could be to readers. | p. 4, lines 107–110  *S1 table added to the submission* |
|  | Methods |  |  |
| 23 | Page 8, line 129: Why was age 18-30 years a inclusion criteria? Why this focus on relatively young adults? | This was an error in the original manuscript. Because our target was young adults, the true age range of the criteria was between 18 and 40 (not 30) years. This information has been corrected in the revised version.  The decision to limit the inclusion criteria to this age range is partly based on the lifespan trajectory of postural control capacity that we mentioned in the introduction (see Lines 52–55 of original version)). We added details to elaborate on our reasons in the participants section of the revision. | p. 5, line 195  p. 5–6, lines 198–199, 208–210 |
| 24 | Page 9, table 2: Table 2 should be presented and described in the result section. The IQ scores of the ID group are quite high, especially because an IQ of 70 (max 75) is indicative of a significant limitation in intellectual functioning. Does this group really represent a group with ID? How was the ID classified, and people with ID selected? | Table has been moved to the results section, as advised.  Indeed the IQ scores fall above the standard cut-off for a diagnosis of ID. However, this can be justified for a number of reasons. First, as stated in the methods (see line 152–152 of original submission), we only used the performance subscale of the abbreviated version of Wechsler, which even if administered completely is only used for screening and research purposes and not for diagnosis. This choice may have overestimated some of the ID-participant’s IQ. Second, all our ID-participants have been recruited from a living facility for people with disabilities (as mentioned in Line 128 of original version) and the nurse provided us with a list of prospective participants which were all diagnosed with ID by a physician. We have added this information in the revision. Lastly, although the margin of error of 5-points is widely accepted, this has been challenged, in particular for low IQ scores (as in the case of ID-individuals). For instance, Whitaker’s^[6]^ review article has estimated this margin of error to be as high as 18 points (8 points above and 10 points below the estimated score).  While it would have been ideal to test them using the full Wechsler Adult Intelligence Scale (WAIS), we did not want to make the test even longer than it already is, as you have also pointed out in your later comment (#30). | p. 9, lines 347–348  p. 10, Table 1 (*former table 2*)  p. 5, line 194 |
| 25 | Page 9, line 139: please add the reference number of the ethical committee to this protocol. | We added the reference number, B322201731833/S59931, in the text as advised. | p. 6, line 213 |
| 26 | Page 9, line 152-153: did you did an assessment with performance subscale of the Wechsler Scale, or was this score reported from for example the participant files? | As indicated in the prior sentence, we could not get access to the participants’ medical records (which included their IQ scores, see lines 151–153 of the original submission). Thus, we implemented the performance subscale of the Wechsler Abbreviated Scale of Intelligence. We emphasized this information to readers by using the verb “administered”. | p. 7, line 269 |
| 27 | Page 9, apparatus and tests: Also report how the other data that was collected; age, sex, BMI, weekly PA hours. All collected data and outcome measures must be described. It will also be useful to describe the outcome measures of the stabilometry with an explanation of them. An average reader, who is not fully aware of these measurements will need an explanation of these outcome measures. | Information added as advised | p. 7, lines 257–261  p. 8, lines 294–299 |
| 28 | Why were these specifications chosen for the measurement protocol (35s trials, 100 Hz sampling frequency)? From the introduction we have learned that a lot of different specifications are used, so please report why these were specifically chosen? Especially, why was the 35s trial chosen, since 60s trails were found to be more reliable? Because one of the aims is to assess which duration would be most suitable and reliable, I would expect that longer trials would have been included as well. | Rationale for 35-s trials have been added as advised  Testing the sampling and cut-off frequency was not part of the study. Thus, we adopted recommendations from Ruhe et al.’s^[7]^ systematic review, which was noted in the text as reference (see line 147 of original submission) | p. 7, lines 266–268  p. 8, lines 299–301 |
| 29 | Page 11, line 220: specify for what comparisons t-test were performed. | We assumed here that you mean Line 202 (because line 220 has no mention of t-test and is in page 12). As advised, we specified, “COP parameters with acceptable reliability as the dependent variable” in the independent sample t-tests that we carried out. | p. 9, line 332–333 |
|  | Results |  |  |
| 30 | The total test duration was quite long, 2hrs. Did people with ID need more rest than the 1 min between trials? | We encountered no problems with the 1-min break between trials. We periodically asked the participants about feeling fatigued. As mentioned in the methods section, we also compared the data to see if the results of the first block systematically differs from the second block (to evaluate fatigue or learning effect) but we found none (see lines 177–180 of original submission). |  |
|  | Discussion |  |  |
| 31 | How representative are these results for the group of people with ID. It is mentioned that results may not be applicable to people with more severe ID, however they may also not be applicable to older adults for the same reasons (worse motor control and skills, more cognitive problems), and perhaps those with moderate levels of ID, behavioral problems etc. The study sample seems to be quite a high functioning group of people with ID (IQ around 75 and quite active) which limits the representativeness to other groups of people with ID, especially since this sample only included 5 participants with ID. This limitation should be made more clear in the discussion. | We explicitly mentioned these points in the text, as advised. | p. 17, lines 501–504 |

**– NOTHING FOLLOWS –**

1. Meshkati Z, Namazizadeh M, Salavati M, Mazaheri M. Reliability of force-platform measures of postural sway and expertise-related differences. J Sport Rehabil. 2011;20(4):442–456. [doi:10.1123/jsr.20.4.442](https://pubmed.ncbi.nlm.nih.gov/22012498/)
2. Smolders C, Doumas M, Krampe RT. Posture and cognition interfere in later adulthood even without concurrent response production. Hum Mov Sci. 2010;29(5):809–819. [doi:10.1016/j.humov.2009.07.009](https://pubmed.ncbi.nlm.nih.gov/19786311/)
3. Hsieh K, Rimmer JH, Heller T. Obesity and associated factors in adults with intellectual disability. J Intellect Disabil Res. 2014;58(9):851–863. [doi:10.1111/jir.12100](https://pubmed.ncbi.nlm.nih.gov/24256455/)
4. Blomqvist S, Olsson J, Wallin L, Wester A, Rehn B. Adolescents with intellectual disability have reduced postural balance and muscle performance in trunk and lower limbs compared to peers with intellectual disability. Res Dev Disabil. 2013;34(1):198–206. [doi:10.1016/j.ridd.2012.07.008](https://pubmed.ncbi.nlm.nih.gov/22944259/)

   Dellavia C, Pallavera A, Orlando F, Sforza C. Postural stability of athletes in Special Olympics. Percept Mot Skills. 2009;108(2):608–622. [doi:10.2466/PMS.108.2.608-622](https://pubmed.ncbi.nlm.nih.gov/19544966/)
5. Horner-Johnson W, Bailey D. Assessing understanding and obtaining consent from adults with intellectual disabilities for a health promotion study. J Policy Pract Intellect Disabil. 2013;10(3):10.1111/jppi.12048. [doi:10.1111/jppi.12048](https://www.ncbi.nlm.nih.gov/pmc/articles/PMC3821759/)
6. Whitaker S. Error in the estimation of intellectual ability in the low range using the WISC-IV and WAIS-III. Pers Indiv Differ. 2010;48(5): 517–521. [doi:10.1016/j.paid.2009.11.017](https://www.sciencedirect.com/science/article/pii/S0191886909004802?casa_token=zZAmvET8ImMAAAAA:sXBp1En1EYv8Iv7pENDZDV08k1rCfx1f6r7HXw4hDZzfwdVcPwYAskBoi1aEy5Q0J5iBqXwpkQQ)
7. Ruhe A, Fejer R, Walker B. The test-retest reliability of center of pressure measures in bipedal static task conditions: A systematic review. Gait Posture. 2010;32:436–45. [doi:10.1016/j.gaitpost.2010.09.012](https://pubmed.ncbi.nlm.nih.gov/20947353/)
